# A Computational Modeling Study of COVID-19 in Bangladesh

**DOI:** 10.4269/ajtmh.20-0757

**Published:** 2020-11-02

**Authors:** Irtesam Mahmud Khan, Ubydul Haque, Samiha Kaisar, Mohammad Sohel Rahman

**Affiliations:** 1 Department of Computer Science and Engineering, Bangladesh University of Engineering and Technology, Dhaka, Bangladesh;; 2 Department of Biostatistics and Epidemiology, University of North Texas Health Science Center, Fort Worth, Texas

## Abstract

The COVID-19 pandemic has spread globally. Only three cases in Bangladesh were reported on March 8, 2020. Here, we aim to predict the epidemic progression for 1 year under different scenarios in Bangladesh. We extracted the number of daily confirmed cases from March 8 to July 20, 2020. We considered the suspected-infected-removed (SIR) model and performed a maximum likelihood-based grid search to determine the removal rate (ɣ). The transmission was modeled as a stochastic random walk process, and sequential Monte Carlo simulation was run 100 times with bootstrap fits to infer the transmission rate (β) and *R*_*t*_. According to the simulation, the (real) peak daily incidence of 3,600 would be followed by a steady decline, reaching below 1,000 in late January 2021. Thus, the model predicted that there would still be more than 300 cases/day even after a year. However, with proper interventions, a much steeper decline would be achieved following the peak. If we apply a combined (0.8β, 1.2ɣ) intervention, there would be less than 100 cases by mid-October, only around five odd cases at the beginning of the year 2021, and zero cases in early March 2021. The predicted total number of deaths (in status quo) after 1 year would be 8,533 which would reduce to 3,577 if combined (0.8β, 1.2ɣ) intervention is applied. We have also predicted the ideal number of tests that Bangladesh should perform and based on that redid the whole simulation. The outcome, though worse, would be manageable with interventions according to the simulation.

## INTRODUCTION

Currently, the world is facing one of the serious pandemics of the world’s history which is caused by SARS-CoV-2.^1^ The current pandemic is referred to as the novel COVID-19 which was first reported in Wuhan Province of China. The transmission of COVID-19 takes place by inhaling the respiratory droplets coming from the coughing or sneezing of an infected person.^2^

It is unknown when COVID-19 spread to Bangladesh. However, the first three cases were reported on March 8, 2020.^3^ To prevent the spread of COVID-19 infections, a nationwide shutdown, in the guise of a general public holiday, went effective on March 26 and continued until May 30.^4^ Currently, 82 laboratories across the country are performing more than 10,000 tests/day.^5^ However, those numbers remained insufficient considering more than 160 million population.

Bangladesh is facing unprecedented challenges to combat COVID-19 because of high population density, a major proportion of people living on their day-to-day income, and the fragile healthcare system. In this study, we provide a forecast of the probable size of the infected cases for the next 365 days, with different intervention scenarios. More importantly, from a public health control perspective, we then forecast the probable course of spread at the national level, considering different interventions (individually or in combination) or no (further) mitigation interventions (i.e., keeping the status quo). This study also predicted alternate intervention and relaxation cycles for 365 days including the total number of patients in the intensive care unit (ICU) at a given time for various combinations of simulations.

## METHODS

### Data source.

The number of daily confirmed cases in Bangladesh was extracted from CSSEGISandData/COVID-19.^6^ We considered the period of March 8, 2020 (first reported incidence) to July 20, 2020 (cutoff date) for this study. We extracted the death rate (among infected patients) from Corona Info Bangladesh^7^ and accessed population data from the Bangladesh Bureau of Statistics.^8^ We also extracted the daily number of tests for all countries from Our World in Data.^9^

### Computational modeling.

#### SIR model.

We used the well-known SIR model (model structure: Supplemental Appendix) for prediction and analysis (Supplemental Figure S4).^10^ In this model, all the population is divided into three compartments, namely, susceptible, infectious, and removed (i.e., isolated, recovered, dead, or otherwise no longer infectious). The whole population of Bangladesh was assumed to be the susceptible population.

In the SIR model, the rates of change from susceptible to infected and from infected to removed are termed, respectively, as the transmission rate, beta (β), and the removal rate, gamma (ɣ). We assumed ɣ to be constant over the period of time because there was not much change in policies regarding testing, quarantine, health care, etc. On the other hand, β was assumed to vary with time because of different intervention policies, social awareness, etc. To find ɣ, we performed a grid search among plausible values thereof known from previous experience. The value found with the maximum likelihood was used in all subsequent analyses. Following the work of Kucharski et al.,^11^ the transmission was modeled as a stochastic random walk process and sequential Monte Carlo (i.e., particle filter)^12,13^ simulation was run to infer β over time. This was run 100 times with bootstrap fits to find out β and *R*_*t*_ (effective reproductive number) over the concerned period of time with different CIs. To be specific, while searching for ɣ, we also found β(*t*) with maximum likelihood. Because our procedure to find β(*t*) depends on the random initialization of β(*t*), we ran the simulation 100 times to generate a distribution of β(*t*) values. In all these simulations, the previously found value of ɣ was used. Our model is based on a random walk process, and if we make the prediction based on the last day’s transmission rate only, it may provide unstable results. But if the trend is captured by taking the average of the last 5 days, the prediction becomes more stable. Therefore, we used the mean transmission rate of the last 5 days for future predictions in all our simulations.

We fitted our model with the number of daily confirmed cases and tried to maximize the likelihood. Informatively, daily recovery data could not be used to fit our model because the guidelines on determining recovered patients changed midway.^14^

We experimented with different scenarios by reducing the transmission rate and increasing the removal rate. We also predicted the number of critical care patients as follows. We considered that a certain percentage of infected patients would need ICU support, and they would stay there for a certain number of days. From this, we predicted the number of patients requiring critical care on a particular day. We also predicted the total number of deaths under different scenarios using the death rate (among infected patients). The total number of deaths was calculated using the following formula:

Total Number of Deaths=Total Infected Person×Death Rate

### Estimating ideal number of tests.

A burning question is whether enough tests are being performed in Bangladesh. Therefore, we estimated how many tests ideally Bangladesh should perform daily. To predict that, we used the following 15 countries as reference: Australia, Belgium, Canada, Germany, France, Italy, the Netherlands, Turkey, the United Kingdom, the United States, India, Spain, Iran, Qatar, and Russia. These countries were selected on the basis of a large number of cases (more cases than Bangladesh) and a greater probability of performing enough tests.^9^ We used the number of new tests performed and the positivity rates (the percentage of positive tests among the tests conducted) in the last 7 days as features and trained a support vector machine^15^ model to predict the number of tests. We used this model to predict the ideal number of tests for Bangladesh. We found the distribution of $\frac{\text{Daily}\;\text{Predicted}\;\text{Tests}}{\text{Daily}\;\text{Reported}\;\text{Tests}}$ and multiplied the median with daily reported cases to find the daily number of ideal cases. This daily number of ideal cases was fed to the same model for further study.

### Reporting probability.

For all practicality, Bangladesh may have more COVID-19 cases (and deaths) than the reported numbers. Because of the lack of appropriate quality data, this cannot be properly modeled. But, to model the epidemic better from this angle, we considered another parameter, namely, reporting probability (δ) that denotes the fraction of the infected population that are confirmed. We made a simplified estimate of the value of δ from the results of a cross-sectional household survey, conducted in the capital between April 18 and July 5, 2020, supported by the United States Agency for International Development and the Bill & Melinda Gates Foundation (The Daily Star, August 10, 2020). The survey indicated that 9% (i.e., 1,800,000) of the total population of Dhaka city was infected by COVID-19, whereas the confirmed cases in that period were 66,000. So, δ can be estimated to be 0.033315. Now, instead of fitting the predicted number of cases (C[*t*]) to the “daily confirmed cases,” we fitted C(*t*) × δ to the “daily confirmed cases.”

### Codes, environment, and availability.

All analyses were conducted using *R* language (version 3.6.3) (Figure 8). We adopted and modified the code provided by Kucharski et al.^11^ To read and manipulate the data, we used *plyr*, *dplyr*, *readxl*, *xlsx*, and *tidyr* along with native *R* packages. For parallelization, we used *mgcv* and *doMC* packages. For plotting data, we leveraged *ggplot2*, *viridis*, etc. packages. All the experiments were conducted in a machine running Ubuntu 18.04.1 with 12 GB RAM and an Intel Core-i5 6200U processor. All codes and data are available in the following Github repository: (https://github.com/rizvi23061998/bd_prediction).

## RESULTS

### Variation of *R*_*t*_.

**Figure 1.**
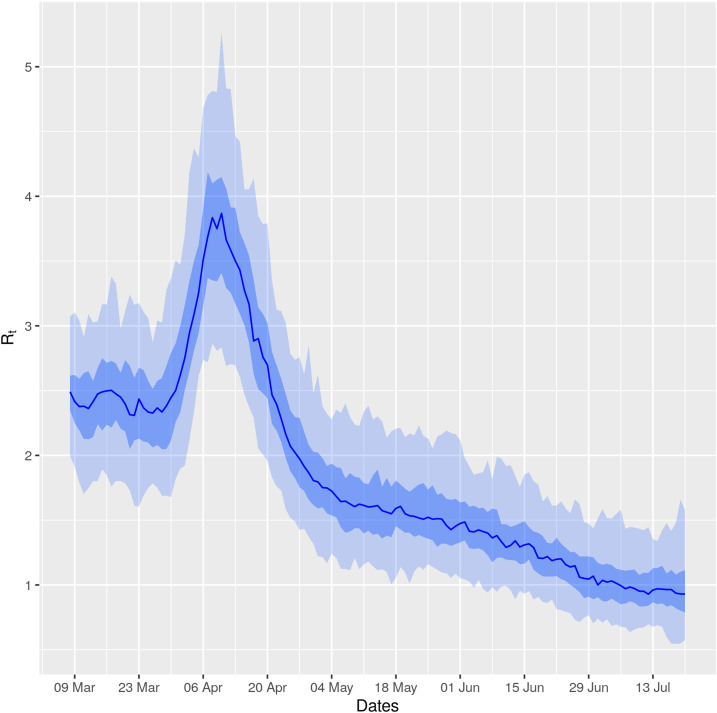
Variation of *R*_*t*_ with time. This figure appears in color at www.ajtmh.org.

Figure 1 presents the variation of *R*_*t*_ with time in Bangladesh for COVID-19 up to July 20, 2020. After an initial rise, *R*_*t*_ seemed to drop a bit during March 20–30, 2020, before rising again. Around April 10–11, *R*_*t*_ reached its highest peak, exceeding four. This peak is then followed by a desirable sharp decline until early May. From early May through June and July, we note a slow, but stable, decline, with *R*_*t*_ reaching a value around 1 (on July 20, 2020, *R*_*t*_ = 0.93).

### Model prediction on status quo.

**Figure 2.**
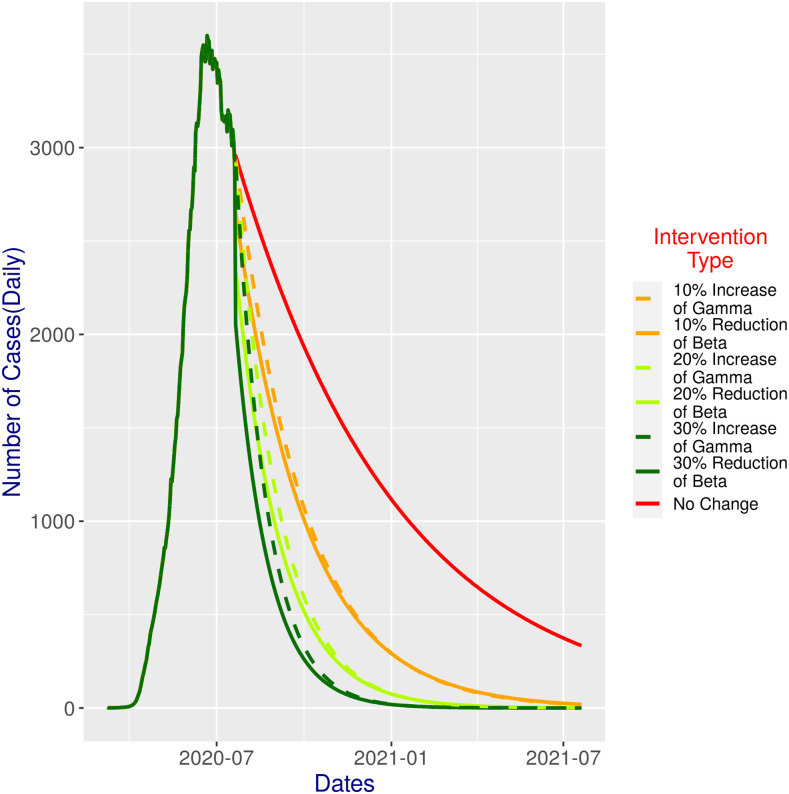
SIR model predictions for 365 days with different (single) intervention scenarios (Table 1) against status quo.

Bangladesh declared a general holiday on March 26, 2020 to implement lockdown. This lockdown was ultimately lifted, after 66 days, on May 31, 2020, principally considering the economic impact and particularly the difficulties faced by people relying on daily works. Subsequently, the government adopted a zoning strategy where local zones were put under lockdown when needed, rather than a nationwide lockdown.^16^ So, the status quo already involves interventions, at least in theory. The model prediction for the status quo (Figure 2) exhibits that the peak was already reached (based on the data) in the third week of June (i.e., on June 21, 2020), with a total of daily incidence reaching 3,600. This would be followed by a steady decline reaching below 2,000 at the end of September and below 1,000 at the end of the third week of January 2021. However, the model predicted that there would still be more than 300 cases toward the end of the simulation, that is, in July 2021, thereby suggesting that a steeper decline would be desirable.

### Intervention scenarios.

#### β and ɣ (individual) interventions.

If β (*ɣ*) can be reduced (increased), we have an impressive effect on the overall epidemic progression (Figure 2). With a 10% reduction of β, we get a slightly steeper decline than the status quo; for example, by the beginning of October, the daily confirmed cases would reduce to below 1,000 (more than the cases 3 months before the status quo). However, the model predicted that there would be around 20 odd cases toward the end of the simulation. If we can ensure a reduction of 20%, the changes would be better: the daily incidence would come down to below 1,000 within one and the half months following the cutoff date, that is, at the end of August, with some odd cases still at the end of the simulation. With a 30% reduction, the reduction would be even better, and we see only around 100 cases from November, and less than 20 cases were predicted by the simulation at the beginning of the year 2021, which gradually would reduce to no cases in early May 2021. Slightly worse effects were predicted for the increase in *ɣ*, for each of the cases (i.e., 10%, 20%, and 30% increases).

#### Combined (β and ɣ) interventions.

**Figure 3.**
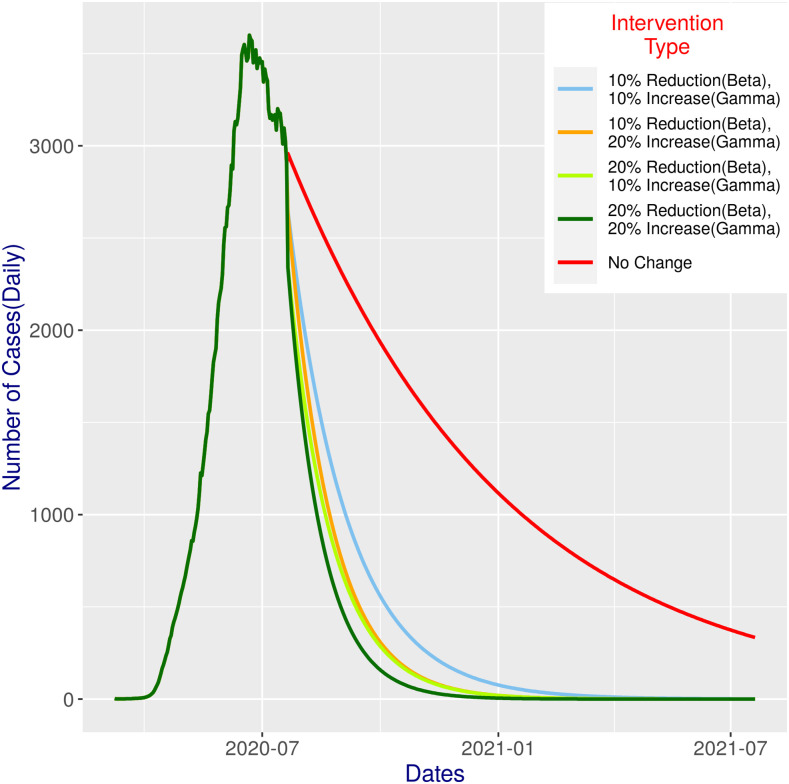
SIR model predictions for 365 days with different combined intervention scenarios (Table 1) against status quo.

As expected, combined interventions show even more promise (Figure 3; Sl. 7–10 of Table 1). As expected, with only 10% change in both, the predicted outcome would be almost identical to the 20% change (i.e., reduction or increase, respectively) on β or ɣ individually. A slightly better outcome would be achieved if we change either ɣ or β by a further 10% (keeping the other same). Of course, if we can reduce β and increase ɣ, both by 20%, we would get an outcome that would be even better than the 30% reduction in β alone (Figure 2): there would be less than 100 cases by mid-October and only around five odd cases at the beginning of the year 2021, which would gradually reduce to no cases in early March 2021.

**Table 1:** Intervention scenarios considered in this study and relevant quantitative results

Sl.	Beta	Gamma	Cases below 2,000 reached	Cases below 1,000 reached	Zero cases reached	Total number of deaths on day 365
0	NC	NC	September 26, 2020	January 20, 2021	NRY	8,533
1	NC	10% increase	August 18, 2020	October 7, 2020	NRY	5,494
2	NC	20% increase	August 8, 2020	September 8, 2020	NRY	4,495
3	NC	30% increase	August 3, 2020	August 26, 2020	May 3, 2021	4,025
4	10% reduction	NC	August 12, 2020	October 2, 2020	NRY	5,324
5	20% reduction	NC	July 29, 2020	August 31, 2020	NRY	4,223
6	30% reduction	NC	July 22, 2020	August 16, 2020	May 8, 2021	3,696
7	10% reduction	10% increase	August 3, 2020	September 5, 2020	NRY	4,363
8	10% reduction	20% increase	July 31, 2020	August 23, 2020	May 5, 2021	3,919
9	20% reduction	10% increase	July 27, 2020	August 20, 2020	May 7, 2021	3,810
10	20% reduction	20% increase	July 26, 2020	August 13, 2020	March 4, 2021	3,577

NC = no change; NRY = not reached in a year.

### Deaths and critical care patients.

**Figure 4.**
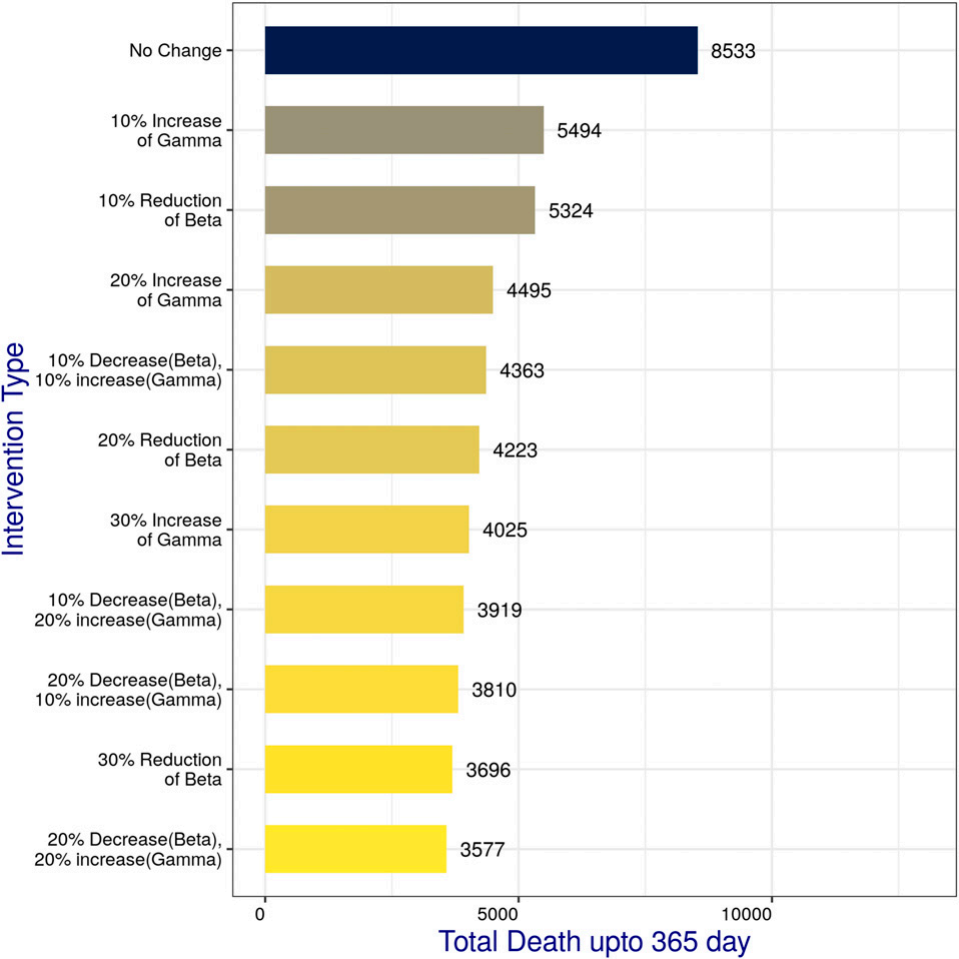
Total number of cumulative deaths on day 365 for various combinations of simulations carried out in this study. This figure appears in color at www.ajtmh.org.

Age-standardized estimates for case severity and fatality of COVID-19 for Bangladesh have been estimated by Chowdhury et al.,^17^ who have reported that the proportion of infected individuals hospitalized is 3.1%, the proportion of hospitalized cases requiring ICU support is 19%, and the proportion of individuals who died requiring critical care is 59%. The overall infection fatality ratio for Bangladesh has been reported to be 0.36.^17^ Bangladesh has a total of 130,437 hospital beds among which ICU beds are only 1,174.^17^ Figure 4 presents the total number of cumulative deaths on day 365 for various combinations of simulations performed in this study. Our model predicted that the status quo, if continued without any further intervention, would result in 8,533 deaths (SQD, i.e., status quo deaths). Noticeably, 30% β reduction would perform quite well with 3,696 deaths (less than half of SQD); even better outcome, that is, 3,577 deaths, was predicted for the combined (β and ɣ) intervention, both at 20%.

**Figure 5.**
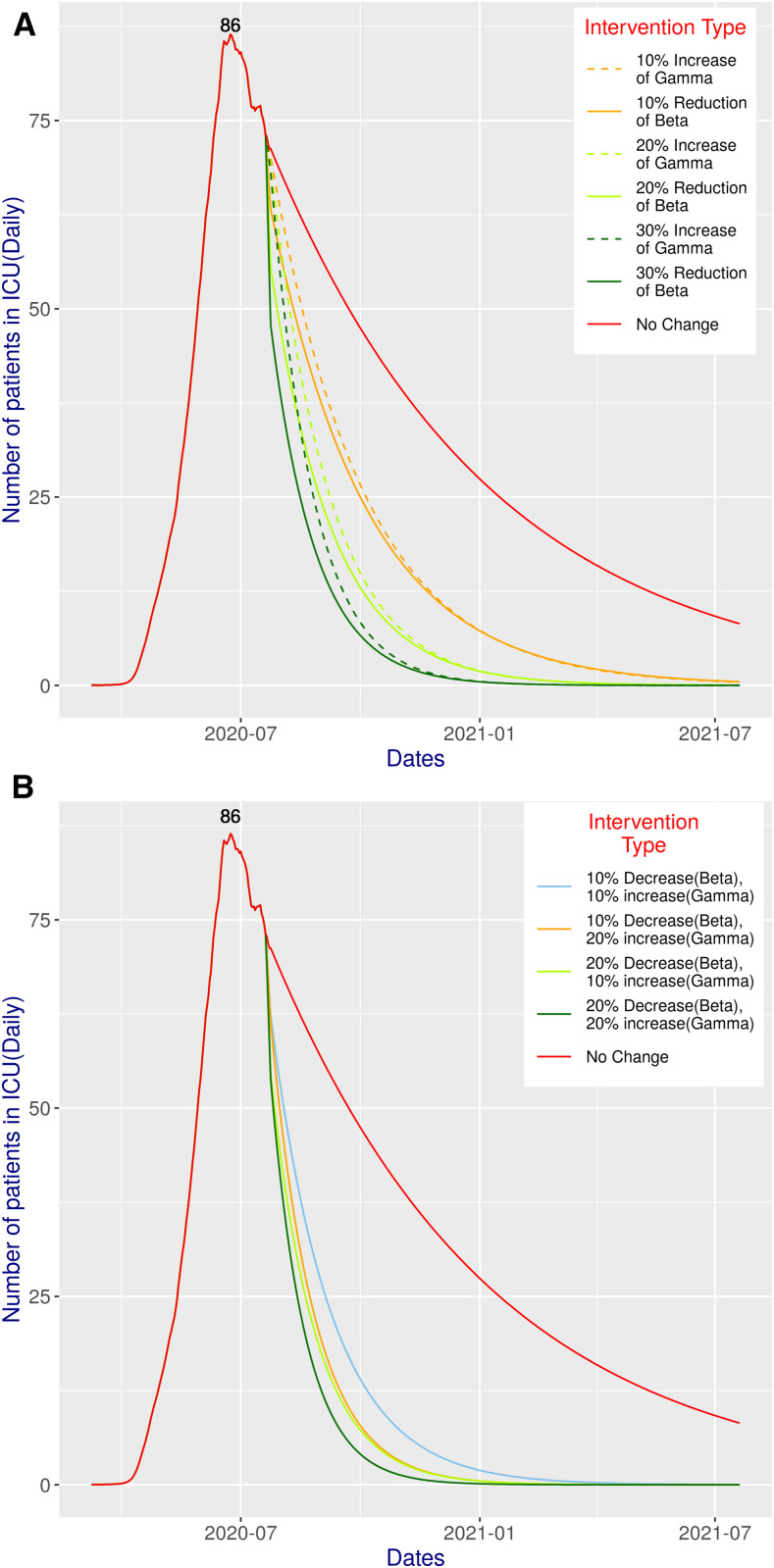
Total number of patients in ICUs at a given time for various combinations of simulations performed in this study assuming an ICU time of 4 days: (**A**) different (single) intervention scenarios; (**B**) combined intervention scenarios.

**Figure 6.**
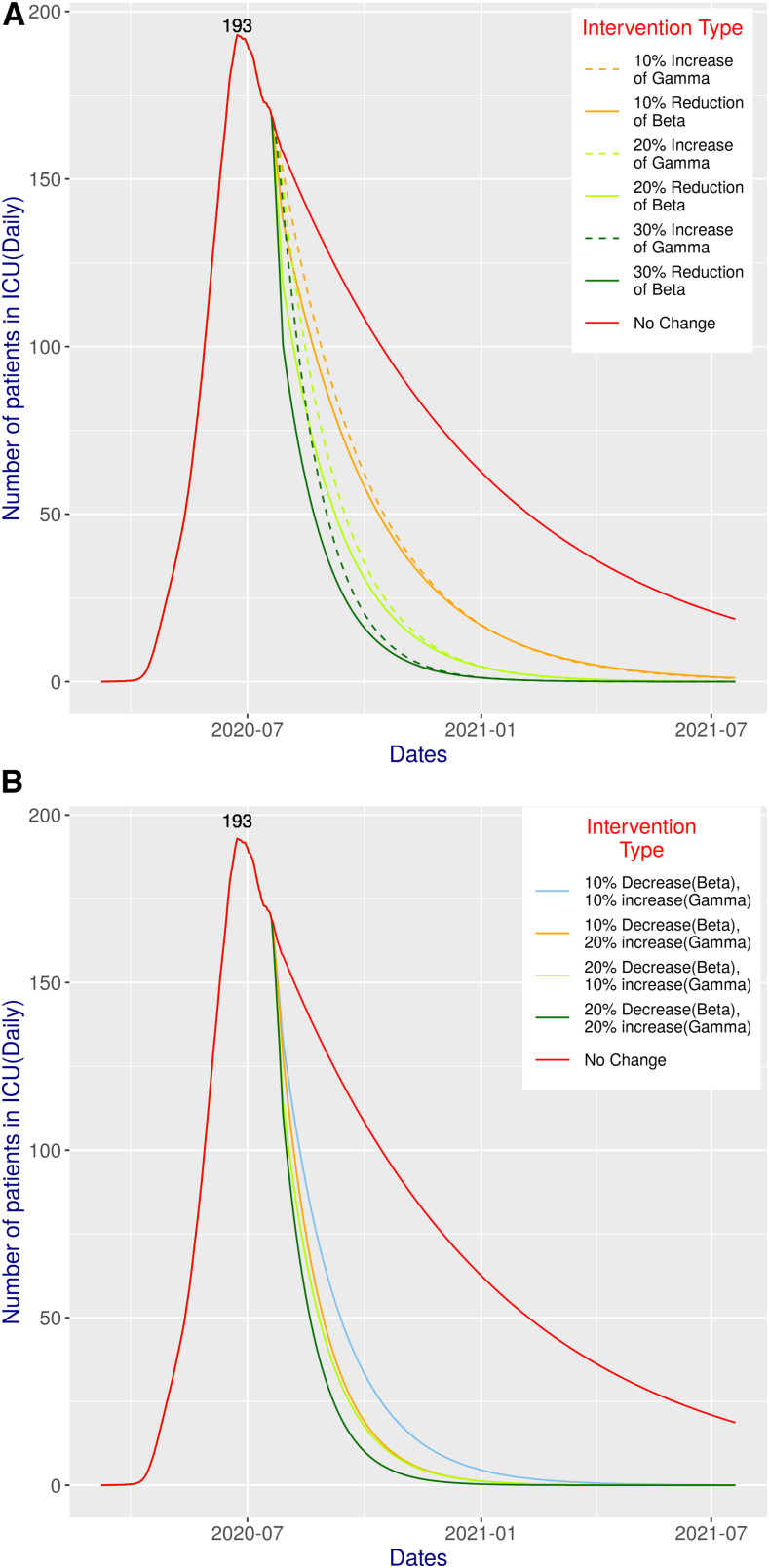
Total number of patients in ICUs at a given time for various combinations of simulations performed in this study assuming an ICU time of 9 days: (**A**) different (single) intervention scenarios; (**B**) combined intervention scenarios.

Figures 5 and 6 present the simulated scenarios of critical care patients for all possible interventions considered in our study. Grasselli et al.,^18^ have shown that the median length of stay in the ICU is 9 days. On the contrary, the Intensive Care National Audit and Research Centre report suggests that the median duration of ICU admission in patients with COVID-19 infections who survive is 4 days.^19^ We simulated considering both, and according to the simulation, the peak value has already been reached on June 24, 2020. With 4 days of ICU duration (Figure 5), the status quo peak value is 86, and with 9 days, it is 193. The corresponding graphs in Figures 5 and 6 actually closely follow the pattern and shape of the curves presented in Figures 2 and 3.

### Ideal number of tests and relevant results.

**Figure 7.**
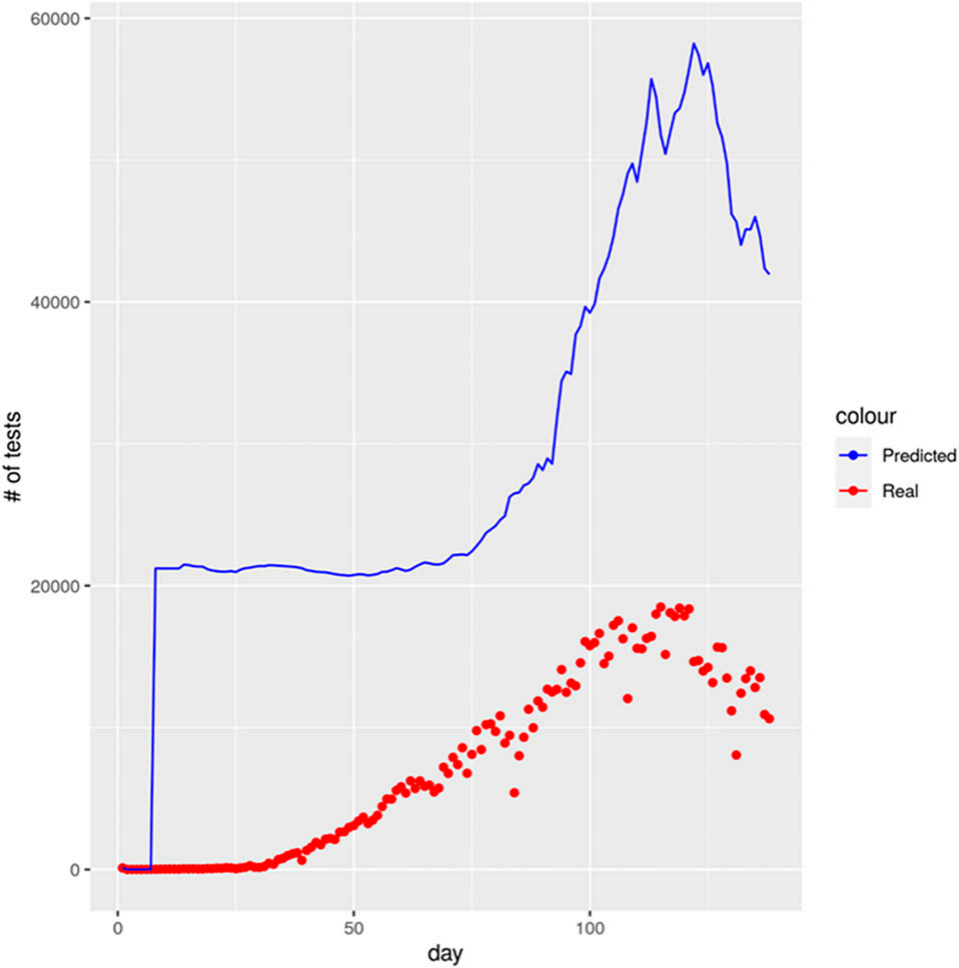
Predicted ideal number of tests for Bangladesh. This figure appears in color at www.ajtmh.org.

The number of tests conducted per day is an extremely crucial factor. Figure 7 reports our predicted estimation (see the Methods section for details) of the ideal number of tests that Bangladesh should have performed. Interestingly, the ratio $\frac{\text{Daily}\;\text{Predicted}\;\text{Tests}}{\text{Daily}\;\text{Reported}\;\text{Tests}}$ was found to have a large range with a median of 3.66. This value suggests that ideally, the number of tests should have been 3.66 times the tests performed. This is understandable, given that Bangladesh has a very high positivity rate (on an average, 15.9% as of July 20, 2020). Evidently, Figure 7 suggests that the number of tests performed at the initial stage of the epidemic should have been a lot more than it was. Based on the previous statement, Supplemental Figure S1 presents a hypothetical scenario where the number of tests has been increased 3.66 times the actual one and consequently the number of cases assumed to be increased accordingly. Understandably, the shape of the curve in Supplemental Figure S1A and B closely follows that of Figures 2 and 3, respectively. According to this simulation, the peak daily confirmed case is 13,588 which had been reached on June 24, 2020. In the status quo, at the end of the simulation, the daily incidence would still be more than 2,000. Although this may sound a bit depressing, the intervention scenarios provide more noticeable improvement now. As expected, the best performer is the combined (β, ɣ) intervention, both at 20% which shows that there would be only five odd cases at the beginning of the year, which would gradually diminish to zero toward the end of February. The total cumulative death count for status quo in this simulation turns out to be 37,681, but with combined β and ɣ intervention, both at 20%, this would be reduced by around 66% (Supplemental Figure S1C). Finally, Supplemental Figure S2 shows the status of critical care patients in this simulation. Notably, even with an ICU time of 9 days, the status quo situation looks manageable, whereas with interventions, the situation naturally would improve a lot.

### Data sensitivity.

The SIR model simulation is highly sensitive to the data fed to it. Also, the prediction is extremely sensitive to the estimation of the *R*_*t*_. This is evident from Supplemental Figure S3 that reports simulation results taking data up to May 24, 2020. Up to that point in time, the value of *R*_*t*_ was estimated to be quite high (Supplemental Figure S3A), and consequently, the curves produced through this simulation in Supplemental Figure S3B are also a vivid reflection thereof (i.e., exceedingly alarming).

### Simulation using reporting probability.

**Figure 8.**
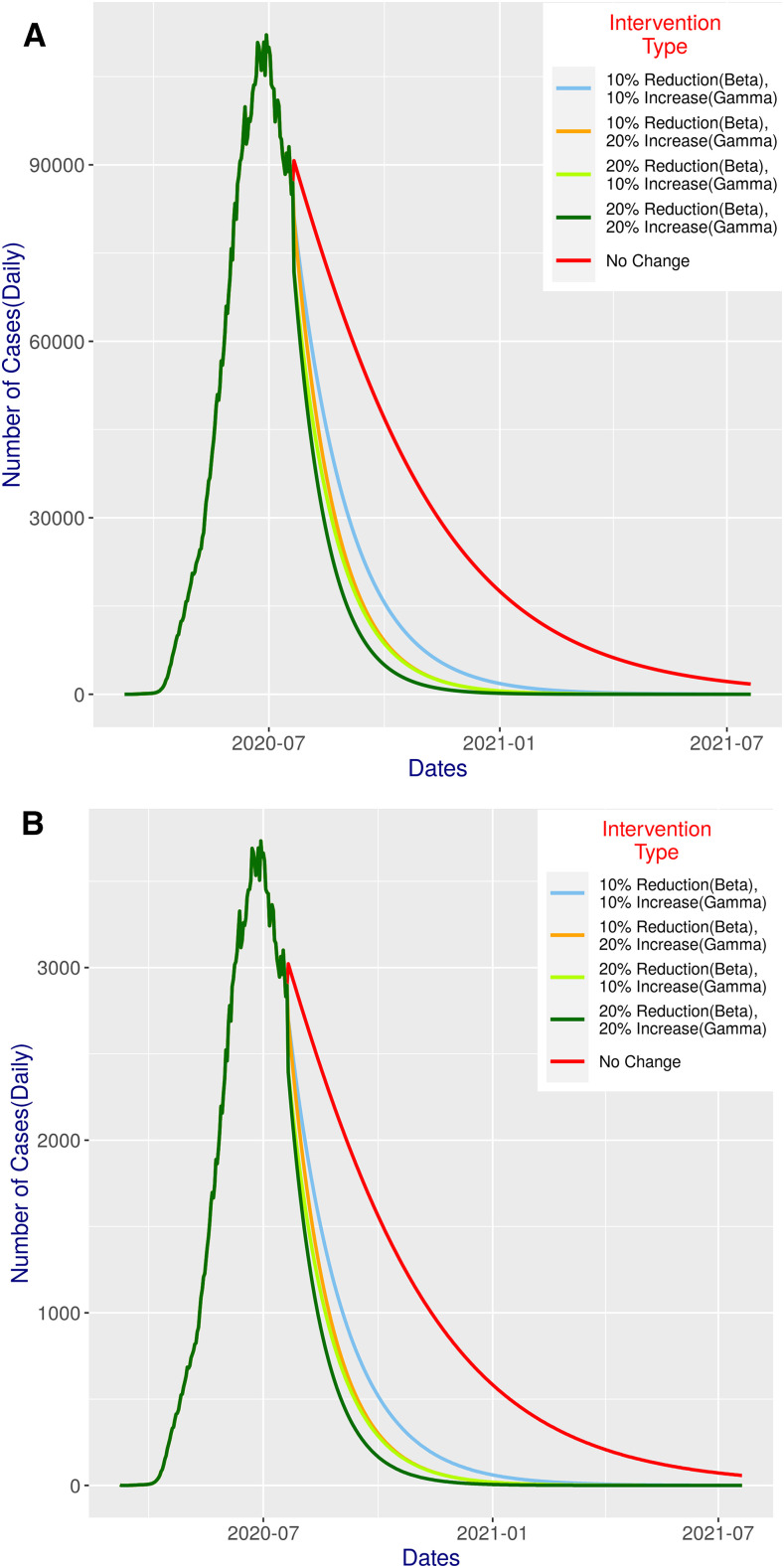
Model predictions with a reporting probability parameter for 365 days with different combined intervention scenarios (Table 1) against status quo: (**A**) the actual cases; (**B**) reported cases.

Figure 8 presents the simulation results with the estimated reporting probability (δ). In particular, Figure 8A presents the actual cases and Figure 8B presents the reported cases. For better visualization, the two figures are not on the same scale. According to this simulation, the actual peak daily confirmed case is 112,091, which had been reached till June 29, 2020. Noticeably, the reported peak daily confirmed case is only 3,734. In the status quo, at the end of the simulation, the actual daily incidence would be more than 1,700, among which only 58 would be reported. The intervention scenarios however show great promise as expected. The best performer is, expectedly, the combined (β and ɣ) intervention, both at 20%. In this case, only five odd cases would be reported at the beginning of the year, which would gradually diminish to zero by the first week of March. However, the actual cases at the beginning of the year would be 164, and the count would not reach zero before the first week of June. In fact, when the reported case becomes zero (i.e., March 7, 2021), there would still be 15 odd cases in reality.

## DISCUSSIONS

The variation of *R*_*t*_ (Figure 1), though looks ordinary, has some intriguing relationship with some real-life events. All educational institutions in Bangladesh were closed since March 17, 2020.^20^ Also around the same time, awareness among people started to build up. The slight drop during March 20–30, 2020, after the initial rise, may be attributed to this. Unfortunately, declaration of general holidays (i.e., lockdown) from March 26, 2020^21^ was not received by the people as a lockdown, rather around one and a half million^22^ people left the capital through intercity transports, which was not suspended until March 26, 2020.^22,23^ This, initially somewhat helped the virus to spread to different parts of the country, resulting in a sharp rise in *R*_*t*_. Also, the highest peak of *R*_*t*_ (during April 10–11, 2020) can be attributed to a mismanagement of the country’s largest trade association of garment manufacturers, when they decided to reopen their factories on April 5, and consequently, on April 4, thousands of garment workers scrambled to reach their workplaces in Dhaka and adjoining areas.^24^ After around a week, the transmission seems to be the highest, and around that time, most of the patients were reported to be garment workers, and Narayanganj, an area famous for garment factories, became a hot spot.^25^

The effective application of interventions indeed seems fruitful (Figure 2). Now, the question is how to map the parametric interventions in the model simulation to real-life ones. Grossly speaking, reducing β is directly related to the enforcement of a proper lockdown and the awareness of people. Other options might be Bangladesh can regularly update the COVID-19 risk maps^26^ and use it to track, quarantine, and implement the lockdown at the mauza level. Risk factor identification,^27^ mapping at the mauza level,^27^ and regularly conducting health behavior study about local people’s attitude and practice and putting them into practice may contribute to effectively control and minimize the risk of COVID-19 spread in Bangladesh. Increasing ɣ directly relates to increasing tests, making infected people strictly quarantined. Thus, this somewhat relates to the medical infrastructure and also has a complex relationship with β: effective quarantine of infected people should reduce β. With financial constraints, it seems more realistic to focus on reducing β (with stricter lockdown enforcement) along with increasing ɣ as much as possible within the budget.

Clearly, with a 30% reduction of β, we would get excellent outcomes (Figure 2). Combined (β and ɣ) intervention at 20% change shows even more promise (Figure 3; compare with Figure 2). The lowest combination (i.e., 10% change in both) would achieve slightly better results than constituent individual interventions. If we decrease β or increase ɣ by further 10%, again the result would be better (albeit only slightly) than all single interventions, except for the 30% reduction in β. Noticeably, the effect of reducing β is more prominent, and hence, more focus could be given for an effective lockdown, thereby ensuring a reduction in β and at the same time increasing the test budget and medical infrastructure if possible. Reducing β and increasing ɣ, both by 20%, would perform even better than the 30% reduction in β as a single intervention.

The death counts for all the combinations of interventions (Figure 4) clearly suggest that the best possible outcome would be (reducing the death count by more than 58% than the status quo) if a combined (β and ɣ) intervention, both at 20%, could be implemented, which is very closely followed by a 30% reduction in β individually. In fact, even the minimum intervention of 10% change in either β or ɣ also would perform well by reducing the death count by more than 35% than the status quo.

Experts have been criticizing that the number of tests performed in Bangladesh is largely insufficient. Newspaper reports and social media outlets continue to report additional deaths attributed to COVID-19 symptoms. Some of the deceased were treated at COVID-19 isolation centers in district hospitals, and others were denied treatment, having received no tests to confirm the contagion. In fact, for a significant period of time in the COVID-19 pandemic, testing was centralized strictly to the Institute of Epidemiology, Disease Control and Research (IEDCR) in Dhaka, the capital of Bangladesh, despite patients reporting COVID-19 symptoms countrywide. With this backdrop, we conducted a simulation where the number of tests was assumed to be *X*-fold (*X* was estimated to be three; Figure 7). The results naturally suggest a worse situation, but the good thing is that the situation can be contained easily with the right interventions. Similarly, a worse situation has been predicted when the reporting probability has been incorporated into the model with a goal to capture the epidemic dynamics better. Even in this simulation, the interventions proved to be quite effective when actual cases (as opposed to reported cases) have been considered.

Our simulation results are highly sensitive to the data used in the simulations. This is clearly evident from Supplemental Figure S3. In particular, if we conduct the same simulation considering data up to May 24, 2020 only, the outcome will look grave. For example, according to that simulation, considering the status quo up to May 24, 2020, the peak would be reached on October 30, 2020, with a total of daily incidence crossing the staggering one million marks (i.e., around 0.8% of the susceptible population). The good thing is that even in that scenario, the right interventions are able to contain the epidemic quite successfully (Supplemental Figure S3B). It may be noted here that, the prediction of peak daily incidence by the model (“Uncontrolled, no intervention scenario”) of Chowdhury et al.^17^ was more than 9 million. The extraordinary difference between our two simulations, that is, the current much improved situation can be attributed to the following: 1) even after the initial hiccups, it seems that the Bangladesh government has been able to implement the right interventional strategies (e.g., zone-wise lockdowns) and perhaps more importantly has been able to instill the right level of awareness among the citizens, and 2) the virus strains responsible for Bangladesh perhaps have become weak or Bangladeshi people are more resistant against the virus.^28^ For example, there is a theory that BCG vaccination could have played a role here.^29^

In light of the previous discussion, it looks imperative that we extend our model to simulate a zone-wise scenario (e.g., to study the effect of the zone-wise lockdown strategy). Unfortunately, the zone-wise data are not available publicly. The gap between the official and unofficial death reporting is not unusual in Bangladesh.^30^ However, the COVID-19 pandemic presents a particularly complex situation; several factors influence death underreporting in the absence of a reporting standard. Members of the civil society have expressed their concern over the country’s current state amid the COVID-19 outbreak. According to the press release, people have questions and doubts over the actual number of deaths and the infected from COVID-19 since the IEDCR announced the first COVID-19 infection in the country on March 8.^31^ These factors include the improper and insufficient testing of COVID-19 patients during their hospitalizations, the time lag between testing and results, and specimen collection inaccuracies.^31^ The concerned citizens have said because the IEDCR has given no opportunity to verify the data are given during the briefings, questions may be raised on accountability and transparency of the briefings.^31^

This study inherits all the limitations of an SIR model. Also because of the lack of data, we could only use a simple SIR model and could not venture for more complex models like the one studied by Giordano et al.^32^ For a similar reason, we could not include the role of asymptotic transmission in our model. Bangladesh may have more cases and deaths of COVID-19 than the reported numbers. This is also not accurately captured in our simplified model. The follow-up attempt to capture this dynamic by estimating the value of δ, that is, reporting probability, also suffers from the limitations imposed by the lack of quality data because the accuracy of this model depends largely on the value of δ. There is no reasonable estimation of δ other than the survey mentioned which was conducted only in Dhaka city. So assuming δ to be the same throughout the country might have been an overestimate. Also, the value of δ may change over the period of time depending on testing policies of the government, public awareness, etc.

Despite all these limitations, this study predicted different scenarios and identified the current limitations to implement the COVID-19 control program in Bangladesh. It will also help other countries to identify a similar limitations Bangladesh faced and address them properly to combat the ongoing COVID-19 pandemic.

## CONCLUSION

We have conducted a computational modeling study of the current COVID-19 scenario in Bangladesh and forecasted for the next 1 year considering different scenarios. We have also incorporated reporting probability into our simplified model to capture the epidemic dynamics better. Our simulation results suggest that with proper intervention, the epidemic progression can be controlled in a graceful manner. An important question is whether the tests being performed by Bangladesh are enough. We developed a model that predicted that the ideal number of tests for Bangladesh should be more than 3-fold. However, our model predicts that even in such a case, the epidemic progression can be controlled with proper interventions. Finally, model prediction on an earlier period of data suggests graver consequences, indicating high sensitivity of the model on the data. Even in such a (hypothetical) case, the impact of interventions was profound. An immediate study must be carried out to determine how the parametric interventions presented in this study can be mapped in real life with appropriate data at the district level. This can only be carried out with a nexus between the government and academia within the country.

## Supplemental appendix, table, and figures

Supplemental materials
