# Supplementary figures and images for: A Computational Modeling Study of COVID-19 in Bangladesh
Source: Am J Trop Med Hyg. 2020 Nov 2;104(1):66–74. doi: 10.4269/ajtmh.20-0757 (PMC7790066; doi:10.4269/ajtmh.20-0757)

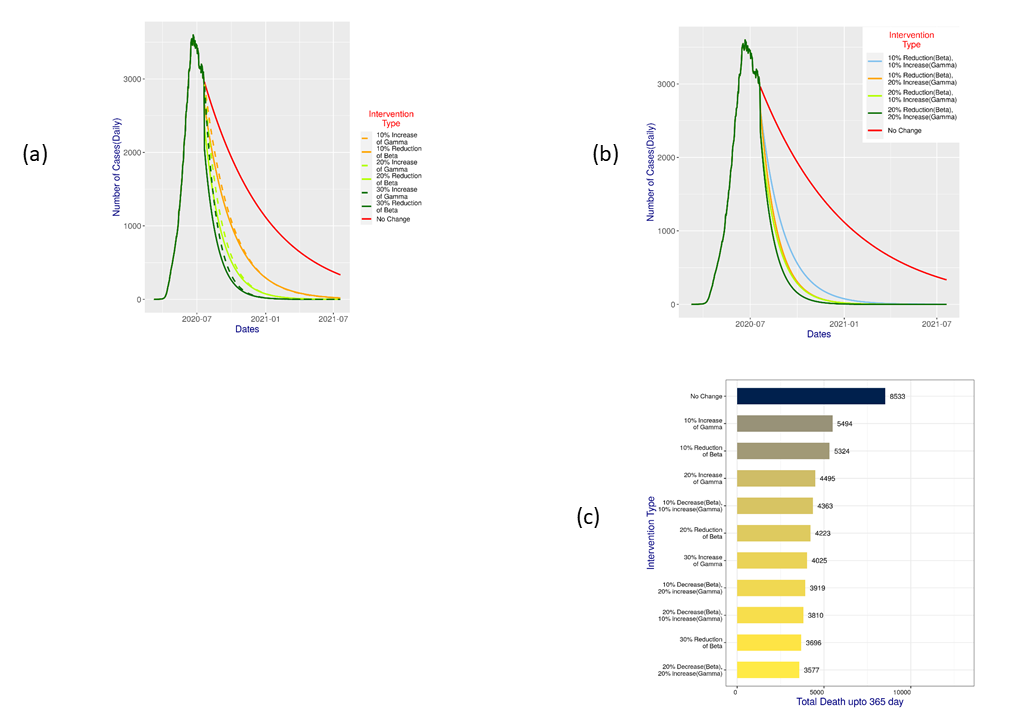

Supplement: Supplementary file 1 [file tpmd200757.SD1.tif]

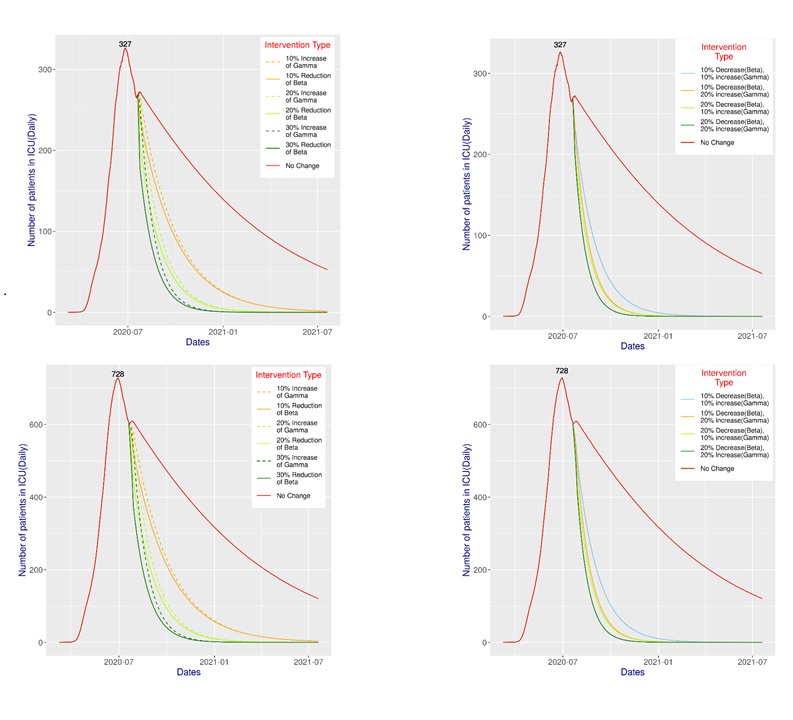

Supplement: Supplementary file 2 [file tpmd200757.SD2.tif]

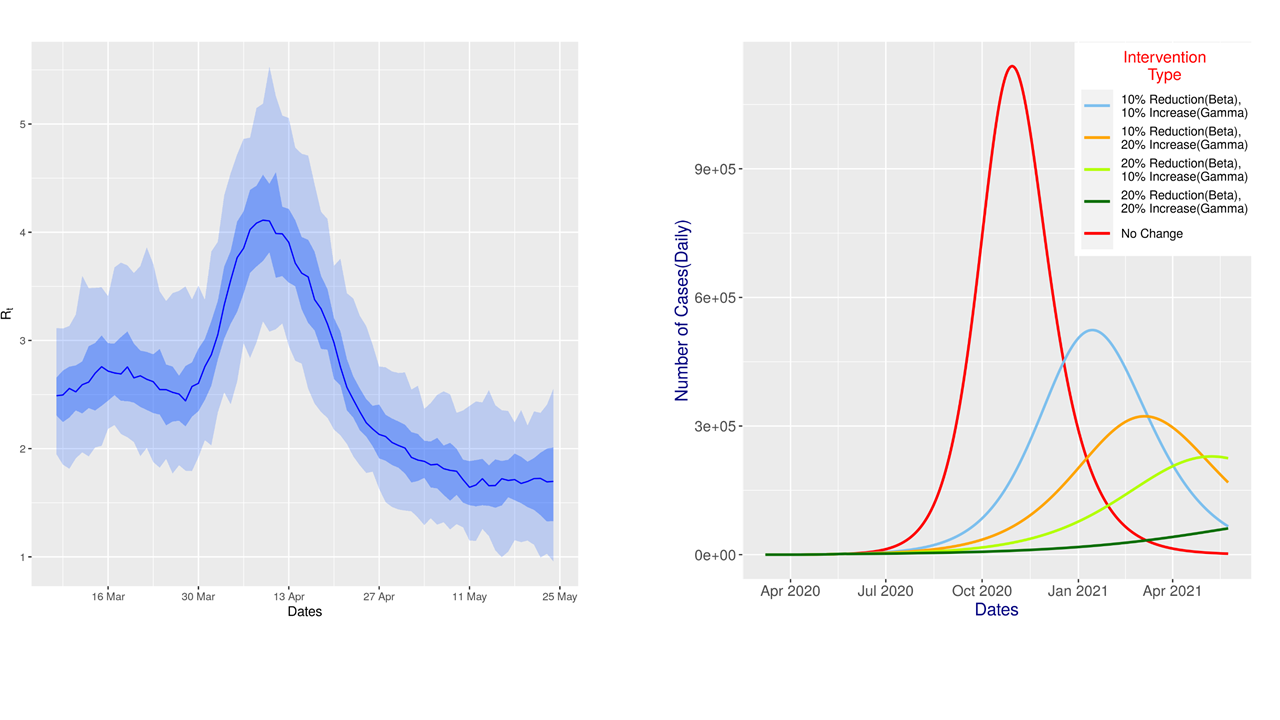

Supplement: Supplementary file 3 [file tpmd200757.SD3.tif]

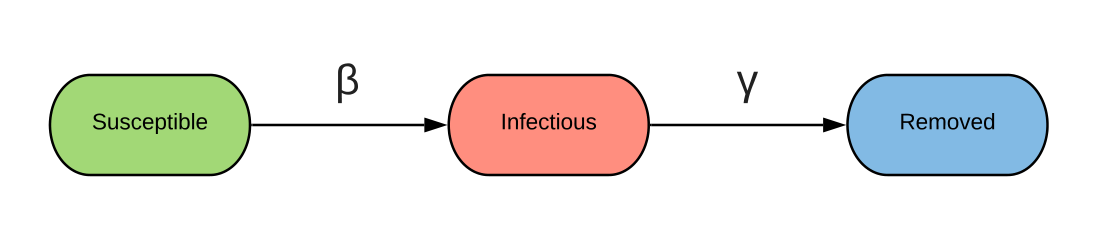

Supplement: Supplementary file 4 [file tpmd200757.SD4.tiff]
