# Supplementary material for: A Computational Modeling Study of COVID-19 in Bangladesh
Source: Am J Trop Med Hyg. 2020 Nov 2;104(1):66–74. doi: 10.4269/ajtmh.20-0757 (PMC7790066; doi:10.4269/ajtmh.20-0757)
Supplement: Supplementary file 5 [file tpmd200757.SD5.pdf]

Supplemental Table S1: Quantitative results for different Intervention Scenarios with predicted ideal number of tests (NC means "No Change" and NRY means "Not Reached in a Year").

Sl.	Beta	Gamma	Cases below 2000 reached	Cases below 1000 reached	Zero cases reached	Total Number of deaths on Day 365
0	NC	NC	NRY	NRY	NRY	37,681
1	NC	10% increase	17/11/2020	03/01/2021	NRY	20,362
2	NC	20% increase	28/09/2020	26/10/2020	NRY	16,130
3	NC	30% increase	08/09/2020	27/09/2020	27/04//2021	14,374
4	10% reduction	NC	13/11/2020	01/01/2021	NRY	19,604
5	20% reduction	NC	21/09/2020	20/10/2020	NRY	15,099
6	30% reduction	NC	30/08/2020	19/09/2020	26/04/2021	13,224
7	10% reduction	10% increase	25/09/2020	23/10/2020	NRY	15,623
8	10% reduction	20% increase	05/09/2020	25/09/2020	27/04/2021	13,999
9	20% reduction	10% increase	02/09/2020	22/09/2020	27/04/2021	13,616
10	20% reduction	20% increase	23/08/2020	07/09/2020	21/02/2021	12,823
