# Supplementary material for: A Computational Modeling Study of COVID-19 in Bangladesh
Source: Am J Trop Med Hyg. 2020 Nov 2;104(1):66–74. doi: 10.4269/ajtmh.20-0757 (PMC7790066; doi:10.4269/ajtmh.20-0757)
Supplement: Supplementary file 6 [file tpmd200757.SD6.pdf]

Supplemental Appendix

Model Structure

Basic SIR model[1] was used to capture the dynamics of COVID-19 in this study (Figure S4). The model was extended to accommodate a class for the cumulative number of cases. Following the work of *Kurcharski et. al.*[2], we used the Euler-Maruyama's numerical scheme for simulation. The stochastic transmission rate, which was modeled as the geometric Brownian motion, was used as the corresponding noise term. The following system of the stochastic differential equation describes the model:

$$S(t+1) = S(t) - \frac{\beta(t)}{N} S(t)I(t)$$

$$I(t+1) = I(t) - \frac{\beta(t)}{N} S(t)I(t) + \gamma I(t)$$

$$R(t+1) = R(t) + \gamma I(t)$$

$$C(t+1) = C(t) + \frac{\beta(t)}{N} S(t)I(t)$$

Here, $S(t) = The\ number\ of\ individuals\ susceptible\ at\ time\ t$

$$I(t) = The\ number\ of\ infected\ person\ at\ time\ t$$

$$R(t) = The\ number\ of\ person\ that\ are\ isolated, recovered, died,$$

$$or\ otherwise\ no\ longer\ infectious\ at\ time\ t$$

$$C(t) = The\ cumulative\ number\ of\ people\ infected\ upto\ time\ t$$

$\beta(t) = Transmission\ Rate\ at\ time\ t$

$$\gamma = Removal\ Rate$$

$$N = Population\ size\ of\ Bangladesh$$

Transmission is modeled as a geometric Brownian motion:

$$d\ log((\beta(t)) = a\ dB_t$$

Here, $a = volatility\ of\ the\ transmission\ rate$

$$dB_t = Brownian\ Motion$$

We simulated the Brownian motion in discrete time by sampling a random number X, which is normally distributed with a mean of 0 and a standard deviation of *a*. Then transmission follows the equation:

$\beta(t+1) = \beta(t)e^X$

Model Fitting

Transmission rate β(t) was estimated using the Monte Carlo method.[3] We calculated the expected value of cases daily from a cumulative number of cases, C(t) using our model. To find the likelihood, we used the PMF of the binomial distribution model with expected values of C(t) per unit time (not cumulative) from the model and evaluated it at Daily Number of Confirmed Cases/Daily Number of Ideal Cases. In the case of reporting probability, we have used C(t) / C(t)δ instead of C(t) for fitting the model.

$\mathbf{R_t}$ is defined as β(t)/ɣ. We generated estimations for β(t) by running 100 repetitions of Sequential Monte Carlo (SMC) with 2000 particles. We selected the removal rate (ɣ) and transmission volatility by performing a grid search among possible values. For example, to estimate ɣ, we initially performed a grid search among 100 possible values X_1, X_2, X_3 … … … X_{100}, where $X_1 = ⅓$ and $X_{100} = 1/20$, and all the other values are linearly interpolated between them. We tried to estimate β(t) for these 100 possible values of ɣ by running SMC with 2000 particles each time. Then we used the value of ɣ with a maximum likelihood to narrow the horizon of our search and perform the same experiment again to estimate a more precise value of ɣ.

For our initial model, we assumed that all of the cases are reported by the IEDCR. The subsequent analysis with an ideal number of cases was done for addressing this limitation.

References

1. Kermack, W. O. & McKendrick, A. G. Contributions to the mathematical theory of epidemics--I. 1927. Bull. Math. Biol. 53, 33–55 (1991).
2. Kucharski AJ, Russell TW, Diamond C, Liu Y, Edmunds J, Funk S, Eggo RM, Centre for Mathematical Modelling of Infectious Diseases C-wg, 2020. Early dynamics of transmission and control of COVID-19: a mathematical modelling study. Lancet Infect Dis 20: 553-558.
3. Del Moral, P. Nonlinear filtering: Interacting particle resolution. Comptes Rendus de l'Académie des Sciences - Series I - Mathematics 325, 653–658 (1997).
